# Unintended Regulatory Caused Early Death—A Difficult Endpoint in Cancer Patient Care and Treatment

**DOI:** 10.3390/cancers13061457

**Published:** 2021-03-22

**Authors:** Wolfgang E. Berdel

**Affiliations:** Department of Medicine A (Hematology, Hemostaseology, Oncology, Pneumology), University Hospital of Muenster, D-48149 Muenster, Germany; berdel@uni-muenster.de; Tel.: +49-251-835-2672

**Keywords:** Good Clinical Practice Guidelines (GCP), regulatory time frames, drug approval and availability, patient safety

## Abstract

**Simple Summary:**

This is a position paper by a clinical oncologist. It voices concerns about political decision making and regulatory time frames for drug availability, both of which are critical for patient care in life-threatening diseases such as cancer.

**Abstract:**

The pharmacological armory against cancer has been growing, with many new drugs approved. The Good Clinical Practice (GCP)-based Clinical Trials Directive was adopted in the EU in 2001, with the important objectives of achieving better patient safety and improved quality of clinical trial conduct. However, clinical experience with the implementation of the regulation raises the question as to whether aspects of this regulatory framework can cause harm to some patients. This question also arises in daily clinical cancer patient care when the time between the publication of pivotal study results and their approval, and details of post-approval regulations, are scrutinized. Clinical observations, provocatively summarized as “unintended regulatory caused early death”, are discussed.

## 1. Introductory Remarks—Historical Perspective

On 1 June 2003, at the Annual Meeting of the American Society for Clinical Oncology (ASCO), some had the privilege to attend a session on innovative clinical trials. Two speakers presented data on survival prolongation for patients with metastatic colorectal cancer (CRC) by two new monoclonal antibodies in combination with chemotherapy. Bevacizumab, targeting vascular endothelial growth factor (VEGF) A (Hurwitz H Proc ASCO 2003, late breaking abstr.), prolonged overall survival (OS), and Cetuximab, targeting epidermal growth factor receptor (EGFR; Cunningham D Proc ASCO 2003, abstracts), prolonged progression-free survival (PFS). The audience listened in silence, countless camera flashes filled the room, and, at the end of the talks, there was fascination and joy. Both trials were published shortly after [1,2]. In the sessions, in which the overwhelming results of the anti-Her2/neu antibody Trastuzumab in women suffering from Her2-positive breast cancer were reported, one could see tears. The leaden decades of the 1980s and 1990s, during which the limitations of conventional chemotherapy against metastasized tumors became so obvious, were past. With novel targeted therapies, progress in cancer treatment started over.

Since then, the pharmacological armory against cancer has been steadily growing, with new drugs approved every year. Few malignant diseases, such as chronic myeloid leukemia or acute promyelocytic leukemia, can today be clinically reduced to a state close to cure. Some metastatic solid tumors can be successfully treated for a prolonged time, with a tolerable quality of life for patients; with the right sequence of therapeutic interventions, we slowly approach the aim of, at least, changing some cancer entities into chronic diseases. That said, there is still an enormous unmet medical need to improve the outcome for patients with advanced cancer.

In 1996, the Good Clinical Practice (GCP) Guidelines were adopted by the Committee for Human Medicinal Products (CHMP) in the EU. The European Union Clinical Trials Directive followed in 2001, again followed by national regulations. GCP was intended to be, and is, an international ethical and scientific quality standard for the design, conduct, performance, monitoring, auditing, recording, analyses, and reporting of clinical trials. This legal framework, although implemented with the objective to improve trial quality and patient safety, has caused tremendous difficulties and delays in the activation and conduct of clinical trials. To this end, the critical literature has pointed to many weaknesses and problems connected with the overregulation of drug development, approval, and distribution [3,4,5]. This opinion paper intends to shed additional light on this area from a clinical oncologist’s perspective. The title and term “unintended regulatory caused early death” was provocatively chosen. The harmful effects of regulation on patient safety are sometimes hidden, not easy to detect, and definitely not as interesting for journalists as the TeGenero accident [6], which filled the lay press. They are not easily scandalized but, nevertheless, can cause an ethical burden for the treating oncologist and investigator. In the following, examples will be given for three areas: drug development within clinical studies, time between publication of pivotal study results and approval, and post approval.

## 2. Good Clinical Practice (GCP) Guidelines—Some Consequences for Clinical Studies

In the experience of the author, the costs of clinical trials in oncology increased 5–10-fold as a consequence of the GCP-caused trial regulation, with large portions of the costs funding centralized supervision–bureaucracy of trials. There is no doubt that GCP guidelines contain indispensable and crucial prerequisites and guidelines for the planning and conduct of clinical studies. Many of those were translated by academic trial groups on a scientific basis before the GCP evolved into an instrument of central legal bureaucracy [7].

Among others, one example of the specific bureaucratic hurdles of the ICH E6 GCP guideline, and the subsequent regulations effective today, is the institution of a “Sponsor”. The Sponsor is responsible for inducing, organizing, and financing a clinical trial, and has transferred the legal responsibility for a trial from the investigators and clinicians to a central institution, and, by this, has enormously increased timelines and costs. For academic investigator-initiated trials (IIT), it is often too expensive to seek a commercial sponsor, and to obtain sponsorship from their institution can take months of hesitation and legal procedures. For example, the cost of a phase I trial in oncology was approx. EUR 50,000.00 before 2001, while, in 2016, it increased to approx. EUR 200,000.00 for academic sponsor-associated activities alone. Sponsor tasks of “quality management and assurance” include monitoring, auditing, and adverse drug reaction reporting. In their actual conduct and detail, for many experienced clinical investigators, these procedures are far from clinical relevance for the trial and its patients and lack evidence for reaching the goal they have been designed for. For example, whereas reporting of suspected unexpected serious adverse events (SUSAR) was performed regularly by direct communication between investigators involved in a specific trial before 2001, today, it is a legally regulated procedure involving written reports to Ethical Boards and higher national authorities, which are often simply not able to check the high number of reports and distinguish between relevant and irrelevant details. Investigators often wonder whether this system is supported by any clear evidence for reaching its objectives of better patient safety and higher trial quality.

In the years after 2001, clinical trials decreased in number [8], and investigator-initiated, non-commercial trials were hit the hardest. Although a causal relation between GCP-caused legal regulation and a decrease in trial activity cannot be easily proven beyond doubt, one example of how a particularly important, but vulnerable, instrument of clinical research in oncology, the so-called “therapy optimization studies” (TOS), has been hit, is in pediatric oncology. Decades of multicenter phase III-type trials improving the algorithms of, often multidisciplinary, cancer treatment by randomized study questions were able to dramatically increase cancer cure rates in children and adolescents [9]. The vast majority of pediatric oncology patients were treated and monitored within these national or international trials—without GCP-caused legal regulation. As in adult oncology, regular phase III trials soon after the implementation of national law, according to GCP, were not financially affordable anymore, and the number of clinical studies performed by the German–Austrian–Swiss Society for Pediatric Oncology and Hematology (GPOH) dropped from 33 in 2002 to 2 in 2017 [10]. At the same time, so-called “clinical registries” increased to 28 [10]. Clinical registries are a reserve instrument to maintain the quality of clinical care by providing centralized structures of data collection and reference evaluation, while circumventing the enormously increased costs of regular phase III trials. Obviously, registries cannot incorporate innovative changes into standard treatment algorithms. Instead, also induced by the new instrument called the Pediatric Investigation Plan (PIP, further details below), the number of drug trials sponsored by pharmaceutical companies has increased. Investigators often wonder whether GCP-caused trial regulation might be a cause for the shift from “academic-” to “pharmaceutical industry-” initiated studies observed in pediatric and in adult oncology, and whether this intention was indeed an important driver for the 2001 regulations.

## 3. From Publication of Pivotal Study Results to Drug Approval

In this journal, Uyl-de-Groot et al. have recently published a detailed study shedding light on “unequal access to newly registered cancer drugs” and how it “leads to potential loss of life-years in Europe” [11]. This investigation sheds light on the delay in approval and, also, on the delay of access in different European countries. In addition, the problem of individual “patient unsafety” in daily oncology practice by rigid regulatory procedures after study results are published has more aspects to be discussed.

Let us again take a historical perspective: when the impact of Bevacizumab, Trastuzumab, Cetuximab, and other targeted drugs meanwhile approved for use in some cancer entities was fully realized, together, with our statisticians, we performed a rough calculation of the events between the detailed report of the randomized trial with Bevacizumab in CRC on 1 June 2003 at ASCO and its regulatory approval in Europe on 25 January 2005. Between these dates, there were approx. 20 months. Assume at this time there were approx. 258,000 new cases of CRC, with approx. 138,400 deaths per year in Europe. Let us cautiously assume that 10% of these patients (*n* = 25,800) fulfilled the main inclusion criteria of the pivotal trial [1]. In these patients, Bevacizumab potentially could have caused an overall survival (OS) prolongation of approx. 4 months [1]. This calculation would mean that 43,000 patients in the 20-month interval had a 4 months shorter OS without the antibody, which adds up to an effect size of 172,000 months, or 14,333 years, of patients’ lives lost earlier due to slow regulatory procedures. Not to be misunderstood, it is a bad idea to skip the relevant control of safety and efficacy for a drug submitted for approval. However, every shortening of approval time, by 50%, in this case, could, hypothetically, have saved 7166 patient-years. It is also clear that this calculation is not completely exact; it does not include confounding factors and cannot be more than theoretical. That said, imagine the destructive avalanche of this effect for oncology when adding up the times, from peer-reviewed publication to approval, of the ever-increasing number of targeted drugs causing OS prolongation in the major neoplastic diseases.

An example in hematology is the history of Gemtuzumab Ozogamicin (GO), an anti-CD33 antibody with the toxic payload, Calicheamicin. The drug was first developed and approved for resistant/relapsed (r/r) acute myeloid leukemia (AML), and was withdrawn from the market by its pharmaceutical company to be re-approved at a later date. It is obvious that the decision making for, and development management of, this drug inside the pharmaceutical industry was far from optimal [12]. However, the French ALPHA-0701-trial was fully published upon peer-review in 2012 [13], trials from the UK were conducted in 2011 [14] and 2012 [15], and a meta-analysis of several trials based on individual patient data appeared in 2014 [16]. This meta-analysis even contained the results of the negative trial of SWOG S0106 using different doses and schedules, which contributed to the earlier withdrawal of the drug from the market in 2010. The evidence for a survival benefit of certain groups of AML patients treated by this drug, in combination with others at that time, was at Cochrane level 1. The results of the ALFA-0701-trial, a GCP-conform, investigator-initiated multicenter study conducted by an experienced AML study group, were taken to the authorities for consultation and were not accepted. Subsequently, the pharmaceutical company had to buy the complete dataset to reevaluate it according to regulatory conditions. Finally, the outcome of this analysis was essentially the same as was published before, and the drug, after 4 years with evidence at level 1 present, was re-approved in September 2017 by the FDA, and in early 2018 by EMA.

Although the meta-analysis [16] also shows some benefit for AML patients with intermediate prognosis cytogenetics, let us assume that GO is only beneficial for patients with cytogenetics known for being favorable. These patients represent approx. 10% of the total AML population [16]. The OS improvement for these patients treated with GO was superior by 20.7% at 6 years, and then survival curves reached a plateau. An AML annual incidence of 3.7/100,000 inhabitants corresponds to 18,870 new AML cases per year in the EU (population 510 million) at that time [17]. Approx. 10% of these patients, i.e., 1887 per year, would benefit from GO therapy if treated according to these results. A 4-year delay of drug approval, after peer-reviewed publication of Cochrane level 1 evidence gathered by major international study groups, corresponds to 7548 patients with a 20.7% worse survival chance at 6 years, i.e., 1562 premature deaths.

Even before the FDA and EMA enforced accelerated or conditional pathways of approval to decrease such devastating effects, there were “compassionate use”, “early access” programs, and similar instruments. These instruments have certainly been helpful but, sometimes, were, and are, imbued with problems, which is illustrated by a brief actual case history observed by the author in 2014: A young patient with acute lymphoblastic leukemia (ALL) relapsed early under standard first-line treatment (GMALL protocol). He was treated within the control arm of a randomized study with one of the promising but, at that time, not yet approved, investigational new drugs (IND) against B-cell precursor ALL, Inotuzumab Ozogamicin (IO). IO induced CRs and CRs with incomplete hematological recovery (CRi) in 57% of patients in this situation in a phase II trial published at that time [18]. After the first cycle of the control chemotherapy regimen, we observed a blast reduction not qualifying for CR, and, after the second cycle, the ALL was refractory. With this clinical course, it was most unlikely that any conventional chemotherapy would yield another remission, allowing us to better proceed to allogeneic stem cell transplantation (allo-SCT) as a potentially curative therapy. The patient had blasts in his marrow and peripheral blood, his general performance was good, and a suitable stem cell donor was available. There was no possibility for the patient to cross over into the IND arm of the study, although the primary objective of the trial was not OS. The Sponsor informed us that the regulatory authorities strongly advised against a crossover design of the study to allow for data acceptance, an advice Sponsors usually follow to not jeopardize IND development. “Compassionate use” of the IND was impossible as long as the patient was in the trial.

What was the ethical concern? There was good evidence that patients without CR have a worse outcome upon allo-SCT than patients in their 2nd or even later remissions (for disease-free survival, e.g., see Doney et al., 2003, Figure 3: 23 vs. 9% [19]; for durable OS see Gökbuget et al., 2012, Figure 4: 39 vs. 20% [20]). There was published evidence that IO induces CR or near-CR in 57% of patients in this situation, with good tolerability. As for regulatory reasons, the IND was withheld for this patient; we had to deny him a possible chance of 57% to receive an allo-SCT in remission, with at least a 10–20% better outcome. Should we have asked the patient to revoke his informed consent? A theoretical chance of obtaining the IND via “compassionate use” might have come too late, study conduct could have been disturbed, and an active drug might have been lost by “friendly regulatory fire”. There was more than one promising ALL IND in randomized trials in this situation and at that time, with similar non-crossover designs although without OS being the primary end point, and refractory ALL is only one example. For our patient, it was an individual problem, but it is indeed a general problem. While pretending to serve “good clinical practice” and “patient safety”, the regulatory system, when dominating trial design and conduct, is potentially life-threatening for individual patients, even if these effects are sometimes hidden by complexity.

## 4. Post Approval

After drug approval, problems are, e.g., caused by “microregulation”, with regulatory restrictions issued together with approval, which, in some cases, at the time of approval or soon thereafter, become obsolete by further scientific evidence. In earlier times, cytotoxic drugs, such as Cyclophosphamide, were more globally approved for the treatment of patients with cancer, while their detailed positioning was left to study groups within the expert scientific community. Today, besides diagnosis, patient groups, lines of treatment, and, sometimes, combination partners and other details are summarized in the approval text. Not following these can cause problems, e.g., reimbursement issues. However, these restrictions also carry risks for patients, as was shown at the ASH 2018 meeting by H. Kantarjian, reporting impressive results of broadening combination partners for the treatment of refractory/relapsing acute lymphocytic leukemia (ALL) by far extending the approved combinations as in the “MiniHCVD-INO-Blina” protocol, which became possible by lowering the doses of combination chemotherapy partners below the level covered by the approval and adding both a targeted antibody Inotuzumab Ozogamicin and a T-cell engager Blinatumomab [21]. With this protocol, a high percentage of r/r ALL patients achieved CR or CRi, with a survival plateau of approx. 30% after 3 years, in many cases after subsequent allo-SCT. Would it not be preferable to leave these and other “microregulations” of treatment protocols to the freedom-to-operate in the scientific hematology/oncology community, with the instrument of peer-reviewed publication of trial results, and stop central bureaucracy before this level?

The above focused on risk–benefit-related procedures. An additional regulatory layer is focused on cost–benefit judgement. Reimbursement control in some countries adds further delays for some patients, which bears the danger of a two-class health system. This is outside the scope of this communication, but our society needs a more open discussion about the problems connected with *rationing* medical therapeutics for patients.

## 5. Problems for Specific Patient Groups

Pediatric oncology is an exemplary area in which regulatory hurdles can lead to disadvantages for specific patient groups. It is a general experience that children tolerate cytotoxic anticancer drugs similar to, or even better than, adults. The majority of conventional cytotoxic drugs in the past had no specific pediatric approval, but were developed by pediatric oncologists, as discussed above, in investigator-initiated multicenter trials within multiagent therapies aiming at “therapy optimization”. This yielded an incredible success story [9]. In recent years a new regulatory instrument, the Pediatric Investigation Plan (PIP), was instituted, which, if there is no specific waiver, is obligatory for drug approval. This certainly has positive aspects, as the scientific data basis for dosing, efficacy, and age-specific tolerability profile has often been insufficient for pediatric patients before. However, one would assume that children suffering from cancer would obtain access to INDs at the same speed as adults; this is not always the case. The development of BiTEs in pediatric ALL, Brentuximab Vedotin in children and adolescent Hodgkin’s disease, and Vemurafenib/Mek inhibitors in adolescent melanoma are examples where regulatory hesitation to allow young individuals on trial has led to substantial delays, e.g., until the individual age cohorts were allowed to be filled by the complete bureaucratic procedure. It is difficult to quantify the effect of this “staggered approach” on possible regulatory-caused early death, but the problem has been recognized by different stake holders [22] and has led to the ACCELERATE platform (http://www.accelerate-platform.eu, accessed on 20 April 2021) to improve the process. Furthermore, the PIP process may even become dangerous for adult patients if it triggers the withdrawal of application dossiers because the requested controlled studies in rare pediatric diseases cannot be performed.

## 6. Recent Developments and Improvements

It is a matter of fairness to acknowledge that drug approval authorities in the US and also in Europe became aware of the problems discussed, and always attempt to adapt and improve pathways within legal boundaries [23,24]. Today, the pharmaceutical industry and authorities cooperate in a much better way; the approval of Ibrutinib with Obinutuzumab in treatment-naïve CLL/SLL by the FDA on 28th January 2019 followed only 2 months after the publication of the iLLUMINATE phase III study on 3rd December, 2018 [25]. Ambitious FDA programs, such as “Real-Time Oncology Reviews” (RTOR), should cross the Atlantic as soon as possible. PRIME, a new program launched by EMA, specifically supports the development of medicines that target unmet medical needs.

## 7. Conclusions and Recommendations

Centrally instituted regulatory hurdles introduced to protect patients from toxicities and ineffective agents may promote unintended early death for cancer patients. More scientific studies should be performed to investigate whether the theoretical benefits of some bureaucratic hurdles in lethal diseases fail to outweigh their dangerous effects. Hurdles without proven positive effects on patient safety and trial quality should be modified or deleted to minimize the danger of patients being caught in the traps described. For instance, EMA could take the initiative to completely revise the ICH E6 GCP guideline, not by the conventional consultation process—in which clinical investigators play only a minor role among the stakeholders consulted—but in a task force with active and, at least, equal participation of physicians who are treating patients on a daily basis in clinical trials, as well as regulators and members of the pharmaceutical industry. Ultimately, it is the politicians within the parliaments and the administrators within governmental structures, in particular, who are asked to translate this into reasonable legal consequences.

## For Transparency

The author is chairman of the joint Ethical Board of the Physicians Chamber of Westphalia-Lippe and the Westphalian Wilhelms University of Muenster.
